# Exploring Consumption of Ultra‐Processed Foods and Diet Quality in the Context of Popular Low Carbohydrate and Plant‐Based Dietary Approaches

**DOI:** 10.1002/fsn3.4496

**Published:** 2024-10-22

**Authors:** Kayla‐Anne Lenferna De La Motte, Jessica L. Campbell, Caryn Zinn

**Affiliations:** ^1^ Human Potential Centre, Faculty of Health and Environmental Sciences Auckland University of Technology Auckland New Zealand

**Keywords:** food quality, food‐classification systems, low carbohydrate, plant‐based, ultra‐processed foods

## Abstract

This study investigates diet quality across four popular dietary patterns: Ketogenic Diet, Low‐Carbohydrate Healthy‐Fat, Vegetarian, and Vegan, employing the NOVA and Human Interference Scoring System (HISS) classification systems. Utilizing a modified Food Frequency Questionnaire (FFQ) and analyzing 168 participants' dietary habits, the research identifies notable differences in dietary quality among the dietary patterns. While all groups reported lower consumption of UPFs than the general population, plant‐based diets demonstrated higher UPF consumption than ketogenic and low carbohydrate diets. The study reveals that both NOVA and HISS effectively identify UPFs, with significant differences observed at various processing levels, except for UPFs where both systems showed similarity. This research contributes to the detailed understanding of diet quality within popular dietary patterns, highlighting the importance of considering food processing in dietary choices and the need for ongoing research to further elucidate the health implications of different types of UPFs.

## Graphical abstract

This study evaluates the diet quality of four popular dietary patterns—Ketogenic Diet, Low‐Carbohydrate Healthy‐Fat, Vegetarian, and Vegan—using the NOVA and Human Interference Scoring System (HISS). Analyzing the dietary habits of 168 participants through a modified Food Frequency Questionnaire, the research found that while all dietary groups consumed fewer UPFs compared to the general population, plant‐based diets had higher UPF consumption than ketogenic and low carbohydrate diets. The study underscores the effectiveness of NOVA and HISS in distinguishing UPFs, highlighting the need for further research on the health implications of UPFs and the role of food processing in dietary choices.

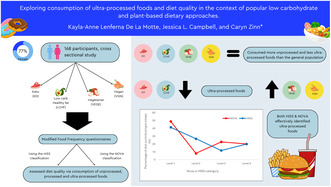

## 1 Introduction

Diet quality is increasingly acknowledged as a critical factor in global nutritional research, serving as a key determinant of health. Recently there has been a shift towards evaluating diet quality not only in terms of nutritional composition but also through the lens of food processing, challenging traditional views and advocating for a more holistic understanding of dietary patterns (Cannon and Leitzmann 2022; Monteiro et al. 2019). Such an approach is particularly pertinent in light of the rising interest in various dietary patterns including Ketogenic (KD), Low‐Carbohydrate Healthy‐Fat (LCHF), Vegetarian (VEGE), and Vegan (VGN) diets (Kamiński et al. 2020). Despite this interest, there remains a significant gap in knowledge regarding the diet quality within these popular diets. The introduction of the NOVA (Monteiro et al. 2019) and HISS (Human Interference Scoring System; Malamatenios et al. 2024) classification systems marks a significant advancement in this area, offering novel insights into the impact of food processing on diet quality. By categorizing foods based on their processing levels, these systems enable a detailed assessment of diet quality across various dietary patterns, crucial for understanding the health implications of these diverse diets.

Alternative dietary patterns are adopted for various reasons, including health, ethics, and environmental concerns, or a combination of the three. While vegan and vegetarian diets have traditionally been adopted due to ethical concerns, more recently meat‐free diets under the umbrella of “plant‐based” diets have been propelled into prominence partly due to the EAT‐Lancet publication, a scientific review outlining a healthy diet from a sustainable food system perspective (Willett et al. 2019). This has led to a significant increase in ultra‐processed food (UPF) formulations catering to this demographic (Willett et al. 2019; Boukid 2021; Wickramasinghe et al. 2021). While a number of health benefits including reduced risk of heart disease, diabetes, obesity, and infectious disease have been associated with traditional plant‐based diets (Campbell 2023; Satija and Hu 2018; Dinu et al. 2017; Qian et al. 2019), the extent to which widely available UPF products impact diet quality and in turn, health, is unclear and remains a topic of debate (Bakaloudi et al. 2021; Bryant 2022; Craig et al. 2021; Melina, Craig, and Levin 2016; Messina et al. 2022). Previous studies have suggested that while well‐planned whole‐food vegan diets meet all micronutrient requirements aside from Vitamin B12 (which is commonly supplemented (Storz et al. 2023; Weikert et al. 2020)), those higher in convenience items do not (De La Motte and Zinn 2023). Similarly, low carbohydrate diets like KD and LCHF which show a spectrum of carbohydrate reduction, have been successfully used to manage medical conditions including diabetes and obesity over long time periods, and, despite previous criticism, have been found to be safe (Athinarayanan et al. 2022; Mckenzie et al. 2022; Roberts et al. 2022). Having been more widely adopted in recent years however, the impact of the growing number of available UPFs is unknown. As with plant‐based diets, micronutrient insufficiency may be more likely in cases where UPFs replace whole foods (De La Motte and Zinn 2023). This research aims to bridge the knowledge gap in understanding the diet quality of popular dietary patterns using the NOVA and HISS classification systems, focusing on the extent of UPF inclusion and its impact on overall diet quality.

The NOVA and HISS classification systems offer distinct approaches to categorizing foods based on processing levels. NOVA classifies foods into four groups, providing a widely used framework for analyzing the impact of food processing on diet quality (Monteiro et al. 2019), while HISS addresses some of NOVA's limitations (Braesco et al. 2022; Sadler et al. 2021; Knorr and Watzke 2019), reflecting the continuum of food processing. The increasing consumption of UPFs, associated with various health concerns (Chen et al. 2020; Costa et al. 2018; Hall 1885; Hall et al. 2019; Lane et al. 2024) underscores the importance of these classification systems in identifying the potential negative impacts of processed foods on diet quality and public health. This research aims to leverage the NOVA and HISS classification systems to assess the extent to which UPFs are included in popular dietary patterns and how this inclusion influences overall diet quality, contributing to a more comprehensive understanding of the implications of modern dietary choices on health.

Utilizing a purposefully developed Food Frequency Questionnaire (FFQ), we aim to explore the relationship between these classification tools and investigate how they correlate with both perceived and actual diet quality. By examining these relationships, the study seeks to provide new insights into the quality of these popular dietary patterns, thereby contributing to the broader understanding of diet quality in the context of modern dietary habits.

## 2 Materials and Methods

### 2.1 Study Design

This study utilized two diet quality classification tools, NOVA (Monteiro et al. 2019) and HISS (Human Interference Scoring System) developed by a team of researchers at Auckland University of Technology's Human Potential Centre (Malamatenios et al. 2024), to assess dietary patterns among KD, LCHF, VEGE, and VGN diets using a FFQ adapted from the 2008/2009 New Zealand Adult Nutrition Survey. While NOVA lacks formal scientific validation but is frequently utilized in diet quality literature, HISS is a newly developed tool, but has been preliminarily validated (Malamatenios et al. 2024). NOVA's application to individual food items versus complex meals presents challenges (Braesco et al. 2022; Sadler et al. 2021; Knorr and Watzke 2019), which HISS aims to overcome by evaluating foods in meal contexts. Using both tools in this work facilitates a comparison of NOVA and HISS's effectiveness and explores whether HISS could be preferred in future dietary quality research. Table 1 outlines the four NOVA and HISS categories respectively with a brief definition of each category and a select number of examples of the foods contained in each. The modified FFQ, designed to capture food processing levels, addresses the critique of using FFQs designed for other purposes (Apovian 2015; Marrón‐Ponce et al. 2018; Moubarac et al. 2017) by leveraging the validated New Zealand survey as a foundation, addressing the gap in tools for assessing processed food intake.

**TABLE 1:** NOVA and HISS category definitions and food examples.

Category	NOVA	HISS
1	*Unprocessed or minimally processed foods* Unprocessed foods refer to the edible parts of plants, animals, fungi, algae, and milk that remain unchanged after being separated from nature while minimally processed foods are those that have been subjected to industrial processing to remove inedible parts of the foods and extend their shelf life. Examples: fresh, frozen, dried fruits and vegetables, whole grains, beans, legumes and pulses, fresh or frozen meat (white or red), poultry and fish, nuts, dried herbs and eggs (fresh, powdered or chilled), milk (fresh, powdered, pasteurized) and fresh fruit and vegetables juices.	*Whole or minimally processed foods* Raw and whole foods that have undergone little to no processing. Processing types in this category include canning, freezing, and drying foods to enhance nutrients and ensure products remain fresh and safe for consumption. This includes foods of plant or animal origin that are processed and stored without additional non‐nutritive substances. Examples: Fresh and frozen raw fruit and vegetables; eggs; red and white meats; fresh, dried, canned beans and other legumes; raw nuts and seeds; honey; fresh, dried, smoked, frozen meat or fish; canned beans, fish, fruit, and tomatoes in spring water; broths; herbal teas.
2	*Culinary ingredients* These are substances in group 1 that have been altered by means of pressing, centrifuging, refining, extracting, or mining and are usually eaten as part of meals rather than alone. Group 2 foods are typically used to season, prepare, and cook group 1 foods. These foods may contain additives to prolong product duration, protect original properties, or prevent proliferation of microorganisms. Examples: Cooking fats (oils, butter, lard), iodized salt, honey.	*Foods mostly available for consumption in pre‐industrial societies* Artisanal products typically available in pre‐industrial, agricultural societies. Products included have been processed using traditional techniques with bacterial (non‐alcoholic) fermentation. Examples: Milk; butter; cheese; milk and coconut creams; unflavored yogurts; artisan bread; coffee beans; rice; pasta; rolled oats; fermented alcoholic beverages such as beer, cider, and wine; spirits; kombucha.
3	*Processed food* Foods that have had group two culinary ingredients added to enhance the flavor, modify, or enhance the sensory qualities of the foods and improve the durability of group 1 foods. Additives to prolong product duration, protect original properties, or prevent proliferation of microorganisms are common in this group. Examples: Vegetables or legumes bottled or canned in brine, smoked or dried meat and fish, salted or roasted nuts and seeds.	*Mostly homemade items* These are foods that have been domestically assembled or hand‐prepared from a collection of group 1 and group 2 ingredients and additional cooking agents as required. As well as foods processed for preservation with additional flavoring and additives with no further cooking needed. Examples: Homemade breads; soups; granola and breakfast cereals; baking and biscuits, peeled or sliced fruit in syrup; canned fish in flavoring or oil; cured meat.
4	*Ultra‐processed foods* These are food formulations, created with five or more ingredients, from items that would not typically be found in a domestic pantry or fridge and using processes that would not be possible in a domestic kitchen. The original component ingredients are not easily identifiable, and products will likely have several additives and preservatives included to prolong shelf‐life beyond traditional processing techniques. Examples: ready‐to‐consume confectionery and convenience products including but not limited to snack bars, chips, pastries and cakes, energy bars, instant drinks, heat‐and‐eat meals like pizza and pies and reconstituted meat products like salami.	*Ultra‐processed food items* Industrially manufactured food formulations that are mass‐produced and packaged ready to eat. The level of processing results in little to no intact whole foods. These food formulations typically contain bulking agents, stabilizers, preservatives, emulsifiers, sweeteners, colors, and flavors to enhance the aesthetics and flavor profile of the foods and ensure they are shelf‐stable for long periods. Examples: reconstituted meats (ham, salami, chicken, and sausage); cured bacon, breads, and wraps; rice cakes; breakfast cereals; biscuits; fizzy and energy drinks; ice cream; sweetened fruit yoghurt; sweetened milk drinks; juice; packaged foods (pizza, burgers, etc.); fries; canned, dehydrated soups; baked beans or spaghetti; muesli bars; protein supplements and bars; cakes; pies; confectionery (chocolate, candy); dressings and sauces; spreads; margarine; baby formulas; ready‐to‐drink alcoholic beverages; instant coffee.

### 2.2 Data Collection

Recruitment was conducted using virtual flyers that were posted on social media platforms (Facebook, LinkedIn, Twitter, and Instagram) by the research team. The Vegan Society of New Zealand and Vegetarian Society of New Zealand shared the virtual flyer with their mailing list. The flyer contained a URL and QR code for convenience. All data was collected anonymously via an online survey tool using Qualtrics^XM^ software between Friday 26th August and Sunday 18th September 2022. All participants read the participant information sheet prior to starting the FFQ and by beginning the questionnaire consented to have their data included in the data set. Participants could exit the survey at any time; incomplete questionnaires were subsequently removed from the final data set. Ethical approval was obtained from AUTEC in September 2022 (approval number: 22/202).

Due to the exploratory nature of this work, there was no required number of participants for the purposes of statistical analysis. However, a convenience sample of 80 participants (at least 20 participants from each diet pattern) was sought. All participants were over the age of 18 years and self‐identified with one of the four popular dietary patterns being explored in this research. Although it is possible that participants may adhere to more than one dietary pattern, for the purposes of this study, they were instructed to select the one they identified with most (ketogenic, low‐carbohydrate healthy fat, vegetarian, or vegan). Participants were included if they were 18 years or older, any sex or gender orientation, and had been eating in accordance with one of the four dietary patterns for at least 6 months. This was important to ensure that they were not new to the dietary pattern and had established a way of eating within the confines of the dietary pattern. Participants were excluded from this research if they were using one of the dietary patterns to lose a significant amount (> 20 kg) of weight as this would likely include a substantial overhaul of diet and lifestyle. Individuals aiming to lose more modest amounts of weight, which likely resulted in more minor modifications of their diet were included. Additionally, individuals who had used one of the dietary patterns as a weight loss tool in the past were not excluded, only those actively seeking to lose substantial amounts of weight at the time that this research was conducted.

### 2.3 Questionnaire Design

A modified FFQ, based on the structure and broad food category groups utilized in the New Zealand 2008/09 New Zealand Adult Nutrition Survey Questionnaire, was developed (New Zealand Ministry of Health, 2008). The FFQ included 82 questions and took participants on average between 15 and 20 min to complete. Participants were asked to consider the number of servings they had consumed, for each question, across a 7‐day period (1 week). The questionnaire was split into four main sections: demographics, food frequency, 24‐h dietary recall, and personal perceptions. Foods were broadly grouped into the following eight subsections: (i) vegetables and fruits, (ii) grain foods and lower carbohydrate substitutes, (iii) dairy products, and dairy substitutes, (iv) legumes, nuts, and seeds, (v) meat, poultry, fish, eggs, and substitutes, (vi) cooking fats, spreads, sauces, and dressings, (vii) confectionery and snacks, and (viii) convenience and takeout. These groupings aligned with the standard Ministry of Health (MoH) groupings and allowed for specialty foods to be explored (e.g., plant‐based meat, plant‐based dairy, and lower carbohydrate bread products). Portion sizes were based on the suggested serving sizes document in the Eating and Activity Guidelines for New Zealand Adults (New Zealand Ministry of Health, 2020). Specialty supplements (e.g., sports performance supplements or herbal tinctures) and vitamins were not included in the FFQ due to their varying quality (and level of processing required for production) of these products and their impact on diet quality remains to be understood.

Once developed, the questionnaire was piloted with a group of six nutrition professionals; their feedback informed amendments to the layout and language used in the questionnaire.

### 2.4 Questionnaire Coding and Dietary Analysis

The response to each question in the FFQ was allocated a NOVA and HISS category number depending on the level of processing the foods grouped in the question underwent. This coding was used to calculate the proportion (as a percentage) of each NOVA and HISS category as it contributed to the overall diet. This was done to apply a quantification element to diet quality without accounting for individual nutrients or energy. Participant occupation was coded using the ISCO‐08 (International Standard Classification for Occupations) structure. The final portion of the questionnaire included questions querying participants about their perceived diet quality and diet adherence; these were assessed using Likert scales (1–10).

### 2.5 Data Preparation and Statistical Analysis

Data were exported from Qualtrics and prepared for analysis in Microsoft Excel. Records for participants who completed < 85% of the survey were removed from the dataset. Following data cleaning, the response to each question was coded to the relevant NOVA and HISS categories. The total number of serves and proportion (%) of total serves was calculated for each participant for each category in NOVA and HISS (i.e., NOVA 1% indicates the proportion of serves in the NOVA 1 group). Finally, diet quality was calculated by dividing NOVA 4% by NOVA 1% (or HISS 4% by HISS 1%); the resultant variable is referred to as N4:N1% ratio (or the H4:H1 ratio). The closer the number is to zero the higher the diet quality and the greater the number the poorer the quality.

For this FFQ, a single serving of takeout (regardless of the size) constituted a single point in the given NOVA or HISS category. While this has been acknowledged as a weakness of food quality classification as information is lost, it is nevertheless a standard approach taken in previous research (Knorr and Watzke 2019; Fangupo et al. 2019).

Data were analyzed using JASP (version 0.16.3.0). Two one‐way repeated measures ANOVAs were carried out to ascertain the difference in the proportion of serves in each level among the diets, first using NOVA classifications and second using HISS. In both cases, data were approximately normally distributed with a few outliers (which were included) but were corrected for non‐sphericity using the Greenhouse–Geisser correction. Post hoc tests were conducted using the Tukey method, and the effect size (*η* ^2^) was calculated. A linear regression was carried out to ascertain whether there was a difference between perceived diet quality and actual diet quality where actual diet quality was represented by the N4:N1 ratio (or the H4:H1 ratio). Both unadjusted and adjusted regressions were carried out, controlling for gender, age, ethnicity, education level, occupation, and income status due to prior research showing such factors may influence nutrition literacy and therefore perceived diet quality (Sanlier et al. 2024). Finally, a two‐way repeated measures ANOVA was executed to ascertain the level of agreement between the two novel tools (NOVA and HISS).

## 3 Results

Table 2 presents the participant demographics for this study. The sample comprised 168 participants, with a majority following low‐carbohydrate diets (56 KD and 66 LCHF) over plant‐based diets (14 VGN and 32 VEGE). Females predominated across all diet groups. Most participants were of European descent, aged 35–64, and had at least a high school education. KD and LCHF respondents tended to be older than VEGE and VGN participants. Demographically, the sample was relatively homogeneous except for diet type and age, with a substantial number working in professional fields and reporting moderate income levels.

**TABLE 2:** Participant demographics presented as number and percentages of the sample in each category.

Demographics	KD	LCHF	VGN	VEGE
Age (years)				
19–24	1 (1.8%)		4 (28.6%)	5 (15.6%)
25–34	4 (7.1%)	2 (3.0%)	5 (35.7%)	8 (25.0%)
35–44	8 (14.3%)	10 (15.2%)	3 (21.4%)	8 (25.0%)
45–54	20 (35.7%)	24 (36.4%)	2 (14.3%)	6 (18.8%)
55–64	14 (25.0%)	21 (31.8%)		
65–69	7 (12.5%)	6 (9.1%)		5 (15.6%)
70+	2 (3.6%)	3 (4.5%)		
Unspecified				
Gender				
Male	16 (29.0%)	13 (19.7%)	4 (28.6%)	5 (15.6%)
Female	39 (70.9%)	53 (80.3%)	10 (71.4%)	27 (84.4%)
Unspecified	1 (1.8%)			
Ethnicity				
African		1 (1.5%)		
Asian	3 (5.4%)	1 (1.5%)		1 (3.1%)
Latin American		1 (1.5%)		1 (3.1%)
Māori			1 (7.1%)	
New Zealand European	23 (41.1%)	39 (59.1%)	8 (57.1%)	20 (62.5%)
Other European	26 (46.4%)	21 (31.8%)	5 (35.7%)	8 (25.0%)
Prefer not to say/ unspecified	3 (7.1%)	3 (4.5%)		2 (6.3%)
Education level				
Trade School or other vocational training	15 (26.8%)	11 (16.7%)	1 (7.1%)	
Primary school			1 (7.1%)	
High school	6 (10.7%)	10 (15.2%)	4 (28.6%)	6 (18.8%)
Bachelor's Degree	21 (37.5%)	25 (37.9%)	4 (28.6%)	21 (65.6%)
Master's Degree	7 (12.5%)	15 (22.7%)	2 (14.3%)	4 (12.5%)
Ph.D. or equivalent	6 (10.7%)	4 (6.1%)	1 (7.1%)	1 (3.1%)
Prefer not to say/unspecified	1 (1.8%)	1 (1.5%)	1 (7.1%)	
Occupation				
Trades/skilled	10 (17.9%)	8 (12.1%)	3 (21.4%)	5 (15.6%)
Professional	33 (58.9%)	45 (68.2%)	7 (50.0%)	17 (53.1%)
Student	1 (1.8%)		3 (21.4%)	3 (9.4%)
Retired	10 (17.9%)	8 (12.1%)		5 (15.6%)
Prefer not to say/unspecified	2 (3.6%)	5 (7.6%)		1 (3.1%)
Unemployed			1 (7.1%)	1 (3.1%)
Income status				
Very low	2 (3.6%)	3 (4.5%)	1 (7.1%)	2 (6.3%)
Low	5 8.9%)	4 (6.1%)	5 (35.7%)	5 (15.6%)
Moderate	28 (50.0%)	30 (45.5%)	8 (57.1%)	18 (56.3%)
High	21 (37.5%)	24 (36.4%)		3 (9.4%)
Very high		3 (4.5%)		2 (6.3%)
Prefer not to say/unspecified		2 (3.0%)		2 (6.3%)

### 3.1 NOVA and Diet Quality

The analysis of NOVA categories among the four diets showed significant differences in dietary intake (Table 3). A one‐way ANOVA highlighted variations in the mean proportions from each NOVA category (henceforth referred to as NOVA%) among KD, LCHF, VGN, and VEGE diets, (*F* (6.150, 336.184) = 8.285, *p* < 0.001), with a medium effect size (*η* ^2^ = 0.063). Post hoc comparisons revealed significant differences in NOVA 1% and NOVA 4% between diets (*p* < 0.001), indicating variations in unprocessed and UPF intake, respectively. Intake of NOVA 1% was significantly higher for the KD group than the VEGE group [diff = 11%, 95% CI 0.02, 0.19] while intake of NOVA 4% was significantly lower in the KD group than either VGN [diff = 18%, 95% CI −0.29, −0.06; *d* = 1.62] or VEGE [diff = 14%, 95% CI −0.22, −0.05; *d* = 1.26] (both *p* < 0.001). Intake of NOVA 4% was also significantly lower in the LCHF group than either the VGN [diff = 14%, 95% CI −0.26, −0.03; 1.33] or VEGE group [diff = 10%, 95% CI −0.19, −0.02; 0.97] (both *p* < 0.001). No differences were found in NOVA 2% or NOVA 3% categories.

**TABLE 3:** Mean proportion of diet from each NOVA and HISS category across the four dietary patterns.

Diet pattern	*N*	NOVA 1 (Mean % ± SD)	NOVA 2 (Mean % ± SD)	NOVA 3 (Mean % ± SD)	NOVA 4 (Mean % ± SD)	HISS 1 (Mean % ± SD)	HISS 2 (Mean % ± SD)	HISS 3 (Mean % ± SD)	HISS 4 (Mean % ± SD)
KD	56	53 ± 12^a^	12 ± 8	21 ± 10	15 ± 12^c^	42 ± 13	32 ± 10^e^	12 ± 8	14 ± 13^g^
LCHF	66	51 ± 13	8 ± 5	23 ± 10	18 ± 12^c^	42 ± 11	28 ± 10	11 ± 8	18 ± 12^gh^
VGN	14	42 ± 18^b^	3 ± 3	22 ± 10	32 ± 51^d^	44 ± 20	14 ± 6^f^	11 ± 8	30 ± 15^hi^
VEGE	32	42 ± 15	4 ± 3	26 ± 8	28 ± 15^d^	37 ± 13	21 ± 8^f^	13 ± 8	30 ± 15^i^

*Note:* Differences between NOVA categories were significant (One‐way ANOVA; *F* = 8.285, *p* < 0.001) with NOVA 1 and 4 being significantly different in post hoc testing. Significant differences in intake of each category between different diets are indicated by letters. Differences between HISS categories were also significant (One‐way ANOVA; *F* = 8.590, *p* < 0.001) with HISS 2 and 4 being significantly different in post hoc testing. Significant differences in intake of each category between different diets are indicated by letters.

These data suggest that, based on this sample, individuals tend to consume a similar percentage of total dietary intake of culinary ingredients and processed foods, regardless of dietary pattern. In contrast, individuals who adhere to a KD tend to eat a significantly greater proportion of unprocessed and minimally processed foods as grouped by NOVA, when compared to VEGE but not when compared to LCHF and VGN. Finally, individuals who adhered to a VEGE or VGN diet tended to consume a significantly greater percentage of total dietary intake of UPF compared to those adhering to either a KD or LCHF.

The main contributors to NOVA 1% for KD and LCHF were meat, poultry, fish, eggs, and substitutes (24% and 13% respectively) and fruit and vegetables (13% and 18% respectively; Table 4). Comparatively, for VGN and VEGE the highest NOVA 1% contributors were fruit and vegetables (27% and 22% respectively). When considering UPFs (NOVA 4) the main contributors for KD were meat, poultry, fish, eggs, and substitutes‐based products (4%) while those adhering to LCHF consumed similar quantities of grain substitutes, and confectionery and snacks (5% each). Comparatively, the highest contributors to NOVA 4% for VGN and VEGE were grains and substitutes (9%) with confectionary and snacks also contributing for the VEGE group (8%).

**TABLE 4:** Mean proportion of NOVA % by food category among the four popular diets.

Food categories	KD mean NOVA % per food group				LCHF mean NOVA % per food group				VGN mean NOVA % per food group				VEGE mean NOVA % per food group			
	N1	N2	N3	N4	N1	N2	N3	N4	N1	N2	N3	N4	N1	N2	N3	N4
Fruit and veg	13		2		18		2		27		4		22		4	
Grains and substitutes	1		1	3	2		2	5	8		1	9	4		3	9
Dairy and substitutes	7		9	1	6		8	2	0		8	3	4		7	3
Legumes, nuts, seeds	3		2	0	6		2	0	6		5	0	6		3	0
Meat, poultry, fish, eggs and substitutes	24		4	4	13		4	3	0		0	6	3		2	3
Cooking fats, sauces and spreads	1	10	3	3	0	8	5	3	0	3	4	5	1	4	5	4
Confectionery and snacks	2		1	3	1		1	5	1		1	6	0		3	8
Convenience and takeout			1	2			1	3			0	4			1	2

*Note:* No displayed percentage for a given NOVA category's corresponding food category denotes that there were no foods allocated to the specific NOVA category in that food group.

### 3.2 HISS and Diet Quality

The analysis of HISS categories among four diets indicated significant dietary intake differences (Table 5). A one‐way ANOVA showed variations in HISS% among KD, LCHF, VGN, and VEGE diets, (*F* (6.912, 377.874) = 8.590, *p* < 0.001), with a medium effect size (*η* ^2^ = 0.086). Post hoc comparisons revealed significant differences in HISS 2% and HISS 4% (*p* < 0.001), with no significant changes in HISS 1% or HISS 3%. Specifically, intake of HISS 2% was significantly higher for the KD group than the VGN group [diff = 18%, 95% CI 0.06, 0.29; 1.60] and VEGE group [diff = 11%, 95% CI 0.03, 0.20; 1.03] (*p* < 0.001 in both cases). Intake of HISS 4% was significantly lower in the KD group than in the VGN [diff = 17%, 95% CI −0.28, −0.05; 1.50] and VEGE group [diff = 16%, 95% CI −0.24, −0.07; 1.42], and was also lower in the LCHF group than the VEGE group [diff = 11%, CI 95% −0.20, −0.03; 1.01].

**TABLE 5:** Mean proportion of HISS % by food category among the four diets.

Food categories	KD Mean HISS % per food group				LCHF Mean HISS % per food group				VGN Mean HISS % per food group				VEGE Mean HISS % per food group			
	H1	H2	H3	H4	H1	H2	H3	H4	H1	H2	H3	H4	H1	H2	H3	H4
Fruit and veg	13		2		14		2		27	4			24		4	
Grains and Substitutes	0	0	2	2	1	0	3	3	6	2	3	7	4	1	4	9
Dairy and Substitutes		16		1		10		2		8		3		13		3
Legumes, nuts, seeds	3		2	0	5		1	0	11	0		0	9		1	0
Meat, poultry, Fish, eggs and Substitutes	24	1	1	5	10	1	1	3	0	0	3	3	3	1	2	3
Cooking fats, Sauces and Spreads		12	1	5		8	0	4		4	0	7		7	1	7
Confectionery and snacks		2	1	3		1	1	4		1	1	6		2	2	9
Convenience and takeout			1	2			1	2			0	4			1	3

*Note:* Where there is no displayed percentage for a given HISS category's corresponding food category, this is because there were no foods allocated to the specific HISS category in that food group.

These data suggest that, based on this sample, regardless of dietary patterns individuals tend to consume a similar percentage of total dietary intake of HISS 1 (whole or minimally processed foods) and HISS 3 (mostly homemade food items) foods. In contrast, individuals who adhere to a KD tend to eat a statistically significantly greater proportion (as % total dietary intake) of foods mostly available for consumption in pre‐industrial societies (HISS 2) when compared to VEGE and VGN but not when compared to LCHF. Finally, individuals who adhered to a VEGE or VGN diet tended to consume a statistically significantly greater proportion of HISS 4 (UPF items) compared to those adhering to a KD, while only those adhering to a VEGE diet consumed a greater proportion of HISS 4 when compared to LCHF.

For the KD and LCHF diets, the primary HISS 1% contributors were animal‐based products, notably meat, poultry, fish, eggs, and their substitutes (24% and 10% respectively), along with a substantial intake of fruits and vegetables (13% and 14% respectively). In contrast, in the VGN and VEGE diets' HISS 1% was dominated by plant‐based items, particularly fruit and vegetables (27% and 24% respectively), and a notable proportion of legumes, nuts, and seeds (11% and 9% respectively). Regarding UPFs (HISS 4%), KDs intake was mainly from animal products (5%) and cooking fats, sauces, and spreads (5%), while LCHF also included confectionery and snacks in addition to similar quantities of cooking fats, sauces, and spreads (4% each). VGN and VEGE diets showed a higher intake from grains and substitutes (7% and 9%) and plant‐based cooking fats, sauces, and spreads (9%), with VEGE additionally consuming significant amounts of confectionery and snacks (9%).

### 3.3 NOVA and HISS Comparison

**FIGURE 1:**
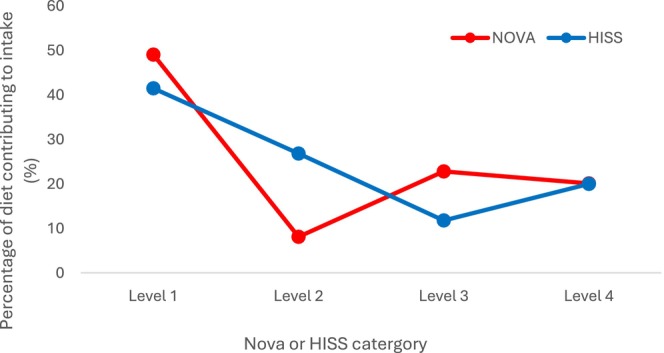
Dietary contribution comparison using NOVA and HISS.

Using pooled data across the four diets there was no difference between the NOVA and HISS diet tools (two‐way repeated measures ANOVA, *p* = 0.754) when data were averaged across all 4 levels. When accounting for the four levels in each system however, there were significant differences between the two tools (Figure 1; two‐way repeated measures ANOVA, *p* < 0.001). The tool * level interaction (*p* < 0.001) suggests the relationship between total proportion (%) intake and the four levels, is different between the two diet quality assessment tools. Post hoc testing revealed the two diet tools were statistically different (*p* < 0.001) at levels 1, 2, and 3, but similar at level 4 (*p* = 0.83), suggesting that both tools are equally capable of recognizing UPFs.

### 3.4 Actual Diet Quality and Perceived Diet Quality

There was a positive relationship between perceived and actual diet quality both when using the N4:N1 ratio and when using the H4:H1 ratio. As results were similar when using either the ratio calculated using NOVA or HISS, only NOVA is presented here (linear regression, *p* < 0.0010, see Data S1 for regression coefficients). As perceived diet quality increased by one unit (on the 1–10 Likert scale), there was a corresponding 0.178 unit decrease in diet quality (DQ) score. Since DQ score was calculated as N4:N1; lower DQ scores reflect diets containing lower percentages of UPFs. The relationship between perceived and actual diet quality can be summarized by the regression equation:

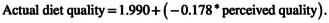




This relationship remained statistically significant after controlling for gender, age, ethnicity, education level, occupation, and income status. Differences between dietary patterns were not formally investigated due to small sample sizes of some groups but the relationship appeared to be strongest for the VEGE group.

## 4 Discussion

Our research critically examined dietary quality in four popular dietary patterns—Ketogenic Diet (KD), Low‐Carbohydrate Healthy‐Fat (LCHF), Vegetarian (VEGE), and Vegan (VGN). Using a modified questionnaire and the NOVA and HISS classification systems, we found notable variations in dietary habits. Notably, both low carbohydrate diets had a higher intake of unprocessed and minimally processed foods though only when using the NOVA system, whereas plant‐based diets demonstrated a higher consumption of UPFs. The NOVA and HISS classification systems showed significant differences at levels 1, 2, and 3, but were similar at level 4 (UPF), indicating that the HISS classification tool is capable of detecting UPFs with comparable accuracy to NOVA, thus supporting the use of HISS for population‐based dietary quality assessments. Finally, we found that there was a positive relationship between perceived and actual diet quality. We suggest that ongoing research is essential to fully understand diet quality when measured by tools including NOVA and HISS but suggest that they offer crucial insights into these popular dietary trends.

### 4.1 Intake Across the Four NOVA and HISS Categories

#### 4.1.1 Unprocessed and Minimally Processed Foods

A key finding of this exploratory work illustrates that (regardless of the measurement tool of diet quality, i.e., NOVA or HISS), all four groups consumed more than 40% of their diet (as a percentage of total serves) from unprocessed or minimally processed foods. For all diet groups, these foods made up the greatest proportion of their diet. This is significant and indicates that those adhering to LC and PB dietary patterns may consume more whole foods than the general population (Machado et al. 2020). Those adhering to a KD or LCHF diet consumed a greater proportion of their diet from unprocessed and minimally processed foods when compared with VEGE and VGN but only when using the NOVA classification system, while HISS group 1 was similar, indicating a difference between the classification systems.

Fruits, vegetables, meat, poultry, and fish were found to be the primary contributors to unprocessed and minimally processed food consumption, aligning with the dietary focuses of the respective dietary patterns. Specifically, LCHF, VGN, and VEGE diets showed significant intakes of fruits and vegetables while the KD diet predominantly included meat, poultry, and fish. This distribution further reflects the dietary preferences and restrictions of each group; KD participants favored higher‐fat and protein foods, while LCHF participants enjoyed a broader range of choices including more fruits and vegetables (which contain more carbohydrate than the more restrictive KD), and PB diets focused extensively on plant foods, as supported by previous findings (Willett et al. 2019; Zinn, Rush, and Johnson 2018). It is notable that although the KD and LCHF groups consumed a higher percentage of foods from NOVA group 1 than the VEGE and VGN groups, the latter two groups consumed more servings of fruits and vegetables. The observed difference was found to be due to higher consumption of animal products in the low carbohydrate and keto groups.

#### 4.1.2 Culinary Ingredients

Culinary ingredients, mainly consisting of fats and oils, formed the smallest portion of the diet for all groups. The KD group exhibited the highest intake of these ingredients, consistent with its low‐carbohydrate, healthy‐fat principles (Zinn, Rush, and Johnson 2018; Volek, Phinney, and Krauss 2021). In contrast, the vegan group had the lowest intake, aligning with their plant‐based dietary preferences and typically lower saturated fat consumption (Bakaloudi et al. 2021; Melina, Craig, and Levin 2016). This pattern reflects the fundamental dietary principles of each diet, demonstrating how distinct nutritional approaches influence the consumption of culinary ingredients. It's important to note that the complexity of assessing these ingredients separately from meals poses a challenge in dietary assessment, a critique of the NOVA system, which the HISS system addresses by not categorizing them separately (Malamatenios et al. 2024; Knorr and Watzke 2019).

#### 4.1.3 Processed Food

We observed that processed foods, classified as NOVA 3, contributed similarly across all four popular dietary patterns. Notably, while the LCHF and KD groups had NOVA 3 as their second largest diet contributor, this was not the case for VEGE and VGN diets. Despite these variations, the overall proportion of processed foods was not significantly different between the diets. This suggests that items typically included in NOVA group 3, such as canned vegetables or freshly made bread, are common dietary staples across different eating patterns. Furthermore, our analysis using HISS, which divides processed foods into two categories (HISS 2 and HISS 3), revealed that KD and LCHF diets had higher intakes of pre‐industrial processed foods (HISS 2) compared to VEGE and VGN.

Given that this is the first study using HISS and NOVA to quantify diet quality, there is a limited body of research on this topic with which to compare these findings. However, when considering NOVA, the findings may be compared to the general population and a small body of research related to PB diets. If diet quality is quantified by the relative contribution of less processed foods, then it could be inferred that LC diets are of a higher quality than that of the general population. This would be expected, given the central tenets of this dietary approach which encourages the consumption of predominantly whole, unprocessed foods (Zinn, Rush, and Johnson 2018). In comparison, the PB respondents consumed a greater proportion of UPF compared to processed foods. Previous research has suggested this could be the result of increased consumption of plant‐based meat and dairy alternatives (Gehring et al. 2021), which may still be interpreted as “healthy” given the health halo around plant‐based foods. However, when items such as tofu are defined as ultra‐processed using NOVA, it is almost inevitable that a vegan diet will be shown to contain more UPFs in all but the strictest sample populations, as discussed below. The extent to which this represents a true decrease in diet quality remains unclear, however. Further work is required to ascertain what the relative contribution of each NOVA or HISS group means for overall diet quality without reducing these holistic frameworks back to a scoring index.

#### 4.1.4 Ultra‐processed Foods

Regardless of the measurement tool of diet quality (NOVA or HISS), the KD and LCHF groups consumed fewer UPFs compared to the VEGE and VGN groups, possibly suggesting lower diet quality in the latter groups. This runs somewhat counter to previous studies finding higher diet quality in vegetarian and vegan diets when compared to a range of other dietary patterns, including ketogenic diets when assessments were based on the Healthy Eating Index (HEI) and the Alternate Healthy Eating Index (AHEI) scores (O'Malley, Willits‐Smith, and Rose 2023). UPFs were a minimal part of KD and LCHF diets, while being more prevalent in VEGE and VGN diets. This trend is in contrast to that of the general population, where UPF consumption is typically higher (Machado et al. 2019; Wang et al. 2022), particularly in Australia, the UK, and the USA (Dicken, Qamar, and Batterham 2023). The fact that those adhering to the popular diets studied consume a smaller overall percentage of their diet from UPFs could be explained by healthy user bias. It is plausible that those following these diets are more concerned about their health, and, as a result, are more selective when making food choices. Furthermore, individuals who perceive their diet as healthy may be more motivated to participate in nutrition‐based research such as this.

The leading contributors to UPF intake varied across diets; while KD diets included more ultra‐processed meat, poultry, and fish, LCHF and VGN diets favored confectionery, while LCHF, VEGE, and VGN groups all included UPF grains and grain substitutes. This is a novel finding in that previous research suggests that the increase in UPF consumption among PB individuals is the result of a reliance on plant‐based meat and dairy substitutes (Gehring et al. 2021). It also suggests that much like PB dietary approaches, through an over‐reliance on novelty and convenience products, LC diets can also be executed with lower overall quality (Gallagher, Hanley, and Lane 2022). While this is yet to be captured in the literature, a simple Google search reveals a community of individuals who align themselves with what is deemed “dirty keto.” This dietary approach typically forgoes food quality with the sole focus placed on achieving the appropriate macronutrient ratios for a KD. As a result, individuals may consume large amount of UPFs as long as they fit within the macronutrient recommendations and nutritional ketosis is achieved, in a similar way to the subgroup of vegans who consume any number of cookies, cakes and chips as long as they do not contain animal products. The impact of this sub‐branch of the KD on overall health is unknown; further research is required to understand if there is a negative consequence to health from selecting UPFs that are low in carbohydrate, which outweighs the benefits derived from carbohydrate reduction alone. We believe that while ultra‐processed grain‐based products and PB meat and dairy alternatives may pose a threat to the diet quality of plant‐based diets, particularly as more vegan products become available, those adhering to LC diets need to be mindful of processed meat products and low‐carbohydrate grain substitutes. These convenient food formulations could increase the risk of metabolic dysregulation via the ingestion of food additives and/or food contact chemicals (Pressman et al. 2017; Muncke et al. 2020) and can cause increased calorie intake and weight gain (Hall et al. 2019). While emerging evidence points to several independent mechanisms by which UPFs could contribute to poor health (Lane et al. 2024), further high‐quality research is required to better understand both their impact on overall health and the reasons for their negative health outcomes.

### 4.2 Comparison of and Critique of Classification Systems

The NOVA and HISS classification systems showed significant differences at levels 1, 2, and 3 but were similar at level 4 (UPF), indicating that the HISS classification tool is capable of detecting UPFs with comparable accuracy to NOVA. Comparisons between NOVA and HISS groups 2 and 3 are however flawed, as the systems are fundamentally different in the way they assign foods to the middle two categories. While NOVA 2 consists of culinary ingredients, HISS does not have an equivalent group, and, consequently, many of the foods that are in NOVA 3 are split between HISS 2 and 3.

Notably, HISS has been validated as an effective tool for food processing classification, as evidenced by recent work (Malamatenios et al. 2024), reinforcing its potential for accurate dietary quality assessments. This validation supports the use of HISS for population‐based dietary quality assessments, though ongoing research is essential to fully understand its applicability across diverse dietary patterns.

We further suggest that HISS attempts to address some of the inherent issues with using the NOVA classification system to assess vegetarian and vegan diets that became apparent during this research. It stands to reason that a vegan diet which excludes animal products, and a vegetarian diet excluding meat and fish would replace them with other sources of protein, often including canned beans and lentils in place of meat. Even before the wide availability of plant‐based meat, tofu and soy milk were common, healthy additions to vegan diets. Under NOVA however, many of these vegan staples are categorized as processed or ultra‐processed, while their non‐vegan equivalents are classed as unprocessed. This categorization means that vegan diets, almost by definition, may appear higher in ultra‐processed and processed foods compared to diets that include meat and dairy, such as diets characterized by carbohydrate reduction. This presents a significant limitation, as it remains unclear whether the types of UPFs consumed by vegans are indeed harmful when in the context of a healthy diet, and more research is urgently needed in this area. Indeed, soy products such as tofu have been widely consumed in some parts of the world over centuries with no evidence of harm, and there is little evidence to show that soy milk is any less healthful than its dairy equivalent (Gardner et al. 2007; Lydeking‐Olsen et al. 2004; Rivas et al. 2002; Jacobsen, Knutsen, and Fraser 1998; Bertron, Barnard, and Mills 1999; Jooyandeh 2011), despite the fact that it may contain synthetic additives. Other UPFs such as plant‐based meat also require more discussion, particularly in light of recent suggestions that they are at least as healthful as unprocessed meat (Crimarco et al. 2022, 2020), highlighting the fact that it is important to consider the net effect of food formulations in the context of a healthy diet, rather than focusing on single ingredients. The current NOVA framework lacks nuance in this regard; the implications of these categorizations for health outcomes are not well‐defined and require further research, as suggested by others (Messina et al. 2022). In part due to these considerations, along with those of participant feedback from another study (Malamatenios et al. 2024) HISS was modified following this work to account for this. More broadly, the assumption that all UPFs are detrimental to health has left NOVA open to criticism (Sadler et al. 2021; Astrup and Monteiro 2022; Drewnowski, Gupta, and Darmon 2020; Vadiveloo and Gardner 2023). Previous studies have not universally found UPFs to be harmful; rather, only specific categories including sugar/artificially sweetened beverages and animal‐based UPFs have been consistently linked to poor health outcomes, while other groups such as yogurts, ultra‐processed cereals, and dark/wholegrain breads have, in contrast, shown inverse associations with chronic health conditions such as type 2 diabetes (Chen et al. 2023). Commercially produced wholewheat bread (NOVA 4) is notable here as UPF grains/grain substitutes were prevalent in all but the KD diets. We suggest that while classification systems based on the level of food processing can be highly informative, there is a need for a more nuanced approach in future research, particularly in examining the health implications of different types of UPFs.

### 4.3 Perceived and Actual Diet Quality

In line with previous research (Powell‐Wiley et al. 2014; Woglom et al. 2020; De Vlieger, Collins, and Bucher 2017), we found a positive relationship between actual and perceived diet quality, with actual diet quality increasing as perceived diet quality increased. This suggests that participants consuming greater proportions of UPFs are at least to some extent aware of the potential negative health consequences and may select these foods for other reasons, possibly including taste, convenience, availability, and/or cost. Previous research has additionally identified that when assessing the “nutritiousness” of snack foods, individuals do consider the level of food processing, alongside other factors such as fat and sugar content and the inclusion of fruits, vegetables, and nuts (De Vlieger, Collins, and Bucher 2017).

### 4.4 Strengths and Limitations of the Study

This research is the first of its kind to quantify the diet quality of four distinct diet patterns and adds new insights to this body of knowledge. A strength of this study was the application of two novel food processing classification frameworks: NOVA, which is well‐recognized in the literature, despite a lack of formal scientific validation, and HISS, a newly developed framework that has undergone scientific validation. This work uncovered similarities between HISS and NOVA at level 4 (UPFs) which will be valuable should either of these classification systems be used in a similar quantification process in future research. This research also resulted in the development of a purposeful FFQ questionnaire to assess and quantify diet quality using the NOVA and HISS frameworks. While it has not yet undergone scientific validation, it does present a new lens and method that can be used to quantify the diet quality of both those adhering to popular diets and the general population, as it uses standardized food groups and serving sizes. Finally, this research developed an index through which to assess overall diet quality (N4:N1% or H4:H1 ratio) using the food processing classification systems of NOVA and HISS. This addition of diet quality metrics adds novel insights into the diet quality field.

The study's limitations are also important to acknowledge. The skewed demographic profile towards European, middle‐aged women of moderate income, particularly in the LC diet group, raises questions about the representativeness of the findings. Age is a significant confounder in our study, especially with the younger demographic prevalent in the vegan group. Younger populations have a higher propensity for consuming UPFs (Dicken, Qamar, and Batterham 2023), which could bias the results regarding UPF consumption in this group. It is possible that a vegan group more comparable in age to the LC groups would have reported lower consumption of UPFs.

There was also an imbalance in the proportion of participants aligning with low carbohydrate compared with plant‐based dietary approaches. This was likely due to most of the social media channels through which the study was advertised being those belonging to two of the researchers, who are advocates of the low carbohydrate pattern. Despite efforts to balance this through engagement with the Vegan and Vegetarian Societies of New Zealand, the resultant small sample size of the vegan group remains a significant limitation which restricts the findings' generalizability and may not accurately represent the broader dietary patterns of the vegan population.

A final limitation of this work is that the study highlights a frequently overlooked bias in dietary motivations between individuals following popular diets. While low carbohydrate or ketogenic diets are typically chosen for health improvement, vegan or vegetarian individuals may or may not be health conscious. Veganism, at its core, is a lifestyle choice which seeks “to exclude—as far as is possible and practicable—all forms of exploitation of, and cruelty to animals” (The Vegan Society 2024) and therefore is not restricted to diet alone. This ethical foundation of veganism suggests that health may not be the primary concern for all vegans, as evidenced by our finding of higher confectionery consumption among vegan participants in the study. Our recruitment didn't exclusively target health‐focused vegans (more accurately individuals who are plant‐based rather than vegan). This difference in the reasons for dietary choices profoundly impacts dietary behaviors and diet quality and we acknowledge that this impacts our comparison between study groups. This aspect is often not sufficiently addressed in dietary pattern research, leading to potential misunderstandings about the dietary habits and health orientations of different groups.

## 5 Conclusions

In conclusion, this study sheds new light on the diet quality associated with various popular dietary patterns, challenging and refining our understanding of these diets, particularly in relation to UPF consumption and its implications for overall diet quality. However, the study's demographic limitations and potential methodological biases underline the need for more comprehensive research in this field. Future research should aim for more diverse participant representation and use validated tools to enhance the accuracy of dietary assessments. This study emphasizes the importance of considering the level of food processing in dietary choices and the crucial role of public health strategies in educating consumers about healthier food selections, irrespective of their dietary preferences.

## Author Contributions

**Kayla‐Anne Lenferna De La Motte:** conceptualization (equal), data curation (lead), formal analysis (lead), investigation (lead), methodology (lead), project administration (lead), resources (lead), writing – original draft (supporting), writing – review and editing (supporting). **Jessica L. Campbell:** writing – original draft (lead), writing – review and editing (lead). **Caryn Zinn:** conceptualization (equal), methodology (supporting), project administration (supporting), resources (supporting), supervision (lead), writing – original draft (supporting), writing – review and editing (supporting).

## Conflicts of Interest

The authors declare no conflicts of interest.

## Supporting information


Data S1.

## Data Availability

The data that support the findings of this study are available on request from the corresponding author.
