# Supplementary material for: Exploring Consumption of Ultra‐Processed Foods and Diet Quality in the Context of Popular Low Carbohydrate and Plant‐Based Dietary Approaches
Source: Food Sci Nutr. 2024 Oct 22;12(11):9651–63. doi: 10.1002/fsn3.4496 (PMC11606827; doi:10.1002/fsn3.4496)
Supplement: Supplementary file 1 — Data S1. [file FSN3-12-9651-s001.docx]

| Regression Statistics | |
| --- | --- |
| Multiple R | 0.487 |
| R Square | 0.238 |
| Adjusted R Square | 0.233 |
| RMSE | 0.580 |
| Observations | 155 |

**Supplementary material**

Table S1. Regression tables for perceived vs actual diet quality using N4:N1. Table shows root mean square error (RMSE), degrees of freedom (df), sums of square (ss), and mean square (MS).

|  | df | SS | MS | F | Significance F |
| --- | --- | --- | --- | --- | --- |
| Regression | 1 | 16.064 | 16.064 | 47.683 | <0.001 |
| Residual | 153 | 51.545 | 0.337 |  |  |
| Total | 154 | 67.609 |  |  |  |

|  | Coefficients | Standard Error | t Stat | P-value | Lower 95% | Upper 95% |
| --- | --- | --- | --- | --- | --- | --- |
| Intercept | 2.380 | 0.267 | 8.908 | <0.001 | 1.852 | 2.907 |
| Perceived diet quality | -0.224 | 0.033 | -6.905 | <0.001 | -0.289 | -0.160 |

Table S2. Regression tables for perceived vs actual diet quality using N4:N1 and adjusting for demographic factors. Table shows root mean square error (RMSE), degrees of freedom (df), sums of square (ss), and mean square (MS).

| Regression Statistics | |
| --- | --- |
| Multiple R | 0.575 |
| R Square | 0.331 |
| Adjusted R Square | 0.299 |
| RMSE | 0.555 |
| Observations | 155 |

|  | df | SS | MS | F | Significance F |
| --- | --- | --- | --- | --- | --- |
| Regression | 7 | 22.349 | 3.193 | 10.370 | < 0.001 |
| Residual | 147 | 45.260 | 0.308 |  |  |
| Total | 154 | 67.609 |  |  |  |

|  | Coefficients | Standard Error | t Stat | P-value | Lower 95% | Upper 95% |
| --- | --- | --- | --- | --- | --- | --- |
| Intercept | 2.623 | 0.343 | 7.650 | <0.001 | 1.945 | 3.300 |
| Perceived diet quality | -0.203 | 0.032 | -6.299 | <0.001 | -0.267 | -0.139 |
| Gender | 0.035 | 0.111 | 0.317 | 0.752 | -0.184 | 0.254 |
| Age | -0.137 | 0.034 | -4.027 | < 0.001 | -0.204 | -0.070 |
| Ethnicity | -0.026 | 0.024 | -1.070 | 0.286 | -0.075 | 0.022 |
| Education | -0.005 | 0.035 | -0.158 | 0.875 | -0.074 | 0.063 |
| Occupation | 0.028 | 0.012 | 2.304 | 0.023 | 0.004 | 0.053 |
| Income status | 0.0165 | 0.050 | 0.330 | 0.742 | -0.082 | 0.115 |
